# Impact of Previous Conventional Cardiac Surgery on the Clinical Outcomes After Heart Transplantation

**DOI:** 10.3389/ti.2023.11824

**Published:** 2023-10-03

**Authors:** Jeng-Wei Chen, Heng-Wen Chou, Nai-Kuan Chou, Chih-Hsien Wang, Nai-Hsin Chi, Shu-Chien Huang, Hsi-Yu Yu, Yih-Sharng Chen, Ron-Bin Hsu

**Affiliations:** ^1^ Department of Surgery, Division of Cardiovascular Surgery, National Taiwan University Hospital, National Taiwan University College of Medicine, Taipei, Taiwan; ^2^ Graduate Institute of Clinical Medicine, College of Medicine, National Taiwan University, Taipei, Taiwan

**Keywords:** heart transplantation, previous cardiac surgery, survival, resternotomy, ventricular assist device

## Abstract

The impact of the type, purpose, and timing of prior surgery on heart transplantation (HT) remains unclear. This study investigated the influence of conventional cardiac surgery (PCCS) on HT outcomes. This study analyzed HTs performed between 1999 and 2019 at a single institution. Patients were categorized into two groups: those with and without PCCS. Short-term outcomes, including post-transplant complications and mortality rates, were evaluated. Cox proportional and Kaplan–Meier survival analyses were used to identify risk factors for mortality and assess long-term survival, respectively. Of 368 patients, 29% had PCCS. Patients with PCCS had a higher incidence of post-transplant complications. The in-hospital and 1 year mortality rates were higher in the PCCS group. PCCS and cardiopulmonary bypass time were significant risk factors for 1 year mortality (hazard ratios = 2.485 and 1.005, respectively). The long-term survival rates were lower in the PCCS group, particularly in the first year. In sub-analysis, patients with ischemic cardiomyopathy and PCCS had the poorest outcomes. The era of surgery and timing of PCCS in relation to HT did not significantly impact outcomes. In conclusion, PCCS worsen the HT outcomes, especially in patients with ischemic etiology. However, the timing of PCCS and era of HT did not significantly affect this concern.

## Graphical Abstract

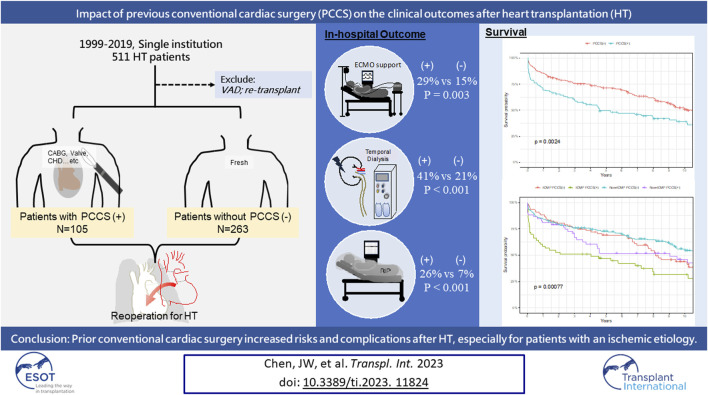

## Introduction

Previous cardiac surgery is a well-known risk factor for increased morbidity and mortality after heart transplantation (HT) [1–5]. Re-sternotomy prolonged the duration of cardiopulmonary bypass (CPB), thereby increasing post-transplant complications, such as coagulopathy, bleeding, infection, acute kidney injury, and acute rejection [3, 6]. Before transplantation, high-risk conventional cardiac surgery cannot be completely avoided, as it still serves as an alternative strategy in cases where the organ is unavailable [7]. However, with advancements in ventricular assist devices (VADs), up to 45% of HTs are performed in recipients who have received mechanical circulatory support before transplantation, and the outcomes have been satisfactory [8–10]. Although VAD implantation also requires an open chest and increases the complexity of subsequent HT, its benefits can mitigate the negative impact of re-sternotomy [11–14]. Moreover, studies have shown that patients who underwent conventional cardiac surgery and subsequently received VAD implantation before proceeding to HT had comparable survival outcomes to those who underwent their first cardiac surgery during HT [13]. With the increased durability of VADs, it remains uncertain whether critically ill patients with heart failure require life-saving conventional cardiac surgery and which type of surgery is warranted. Furthermore, it is unclear whether the impact of prior cardiac surgery on HT has changed, given advancements in perioperative care and growing experience with re-sternotomy. This study exclusively focused on investigating the impact of different types, timings, and operative eras of previous conventional cardiac surgery (PCCS) on the outcomes of patients undergoing HT within our hospital.

## Patients and Methods

**FIGURE 1:**
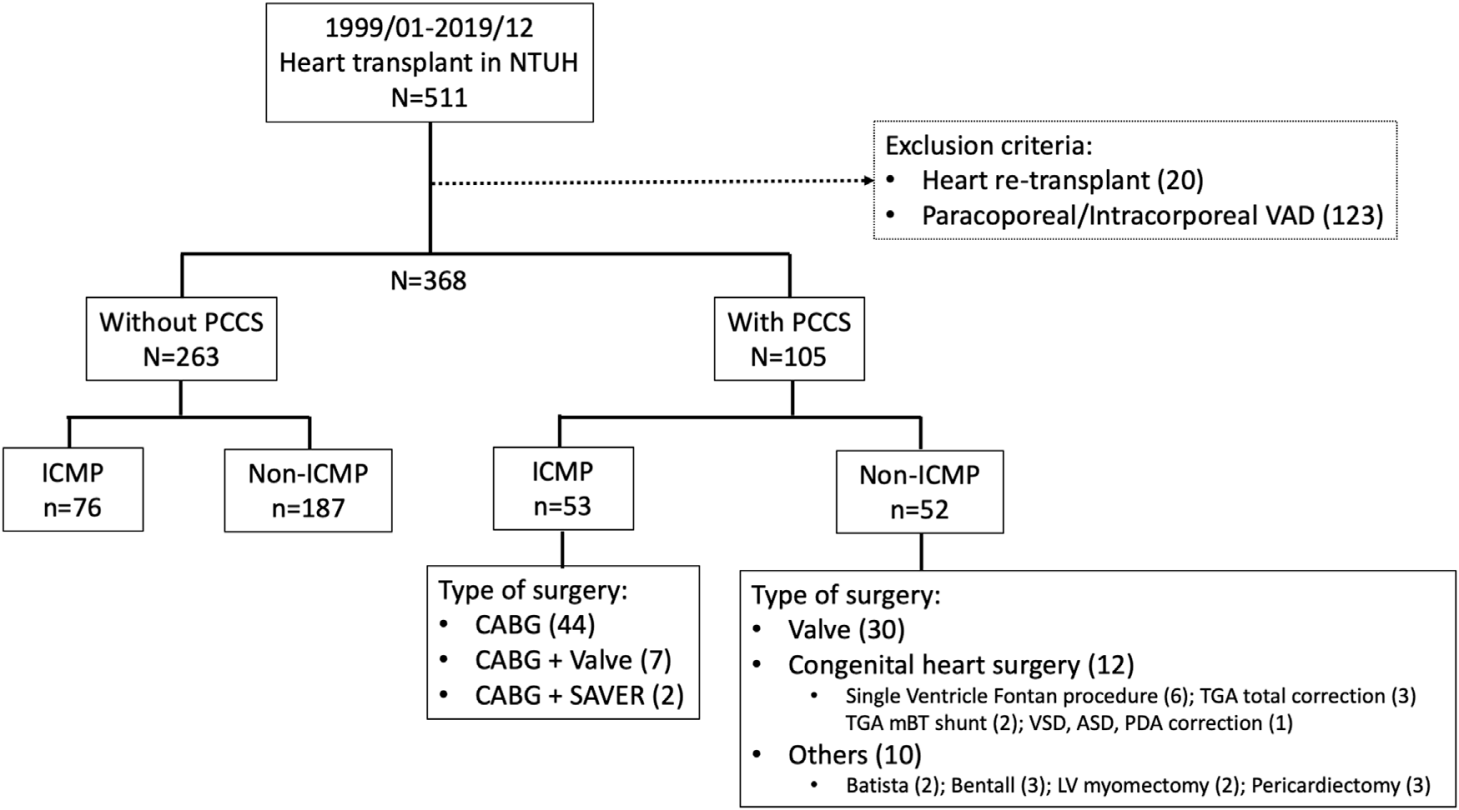
Details regarding the group and type of previous conventional cardiac surgery in study patients. CABG, coronary artery bypass surgery; ICMP, ischemic cardiomyopathy; PCCS, previous conventional heart surgery; SAVER, surgical anterior ventricular endocardial restoration.

All HTs performed between January 1999 and December 2019 at the National Taiwan University Hospital were included in the study. This study was approved by our Institutional Review Board, and the requirement for informed consent was waived (202208017RINB). Data were collected through a retrospective chart review of a prospectively observed patient cohort. Our hospital conducted the initial HT in 1987, followed by the first VAD implantation as a bridge to HT in 1997. Taiwan’s national health insurance has covered paracorporeal VAD since 2011 and intracorporeal durable VAD since 2018. In this study, we excluded patients who were bridged to HT with a VAD and those who underwent heart re-transplantation because of allograft dysfunction (Figure 1). Other cardiac surgeries, apart from those stated earlier, were recognized as conventional cardiac surgeries. The rationale behind this exclusion is that VADs serve as alternative tools to stabilize patients and potentially improve the outcome of HT, which introduces selection bias [8, 9]. Furthermore, re-transplantation for allograft dysfunction is known to have a poor prognosis because of immune sensitization [15, 16]. All patients were categorized into “with PCCS” and “without PCCS,” based on whether they had undergone conventional cardiac surgery before HT. The primary outcomes assessed were the short- and long-term survival rates. Secondary outcomes included postoperative morbidities, such as re-exploration or delayed sternum closure, renal dialysis, early bloodstream infection (within 30 days), and post-transplant hospital stay. The etiology of heart failure and the type of cardiac surgery are highly associated. To evaluate the impact of the initial operation, both groups were further divided into two subgroups based on the purpose of surgery: PCCS for ischemic cardiomyopathy (ICM) and non-ICM. Patients were divided into two subgroups to investigate the timing of PCCS in relation to HT: over 2 years or within 2 years, depending on the patient distribution. Additionally, both groups were divided into two subgroups based on the year of HT in our hospital (1999–2009 and 2010–2019) to examine the impact of the new era compared with the old era. While the allocation system in the United States was expanded in 2018 to include seven statuses, designed to address the diverse situations of VAD-supported HT candidates, our study retained the prior allocation framework. We specifically focused on United Network for Organ Sharing (UNOS) statuses 1A, 1B, and 2, as we excluded VAD patients from our study.

### Management of Patients With PCCS Receiving HT

When enrolling patients who have undergone PCCS and are currently receiving HT, several important points need to be considered. We routinely performed pericardial closure during the initial surgery or used anti-adhesive patches when pericardial tissue was insufficient. Preoperative CT for re-sternotomy risk assessment was always performed. Although it may not be possible to cease anticoagulation and antiplatelet medications before HT owing to the unpredictable timing of organ availability [17], we promptly evaluated the candidate’s medication profile and initially suspended any potentially harmful drugs. As a preparatory measure before re-sternotomy, we routinely exposed the femoral artery and vein as an emergency route for CPB setup. To avoid the need for emergent CPB, it is crucial to allow an adequate amount of time for the surgeon to perform dissection. Continuous communication between the donor organ harvest team and the recipient preparation team is necessary to minimize CPB and allograft ischemia time. After confirming the suitability of the donor heart, the recipient team performed re-sternotomy. In urgent situations such as unexpected bleeding or changes in the donor’s condition, rapid CPB is established via femoral access for quick heart decompression and re-entry. After surgery, sternal closure may be delayed for 24 h if adequate hemostasis is not achieved. Furthermore, immunosuppressant and desensitization protocols were followed as usual, based on previous publications [18–21].

### Statistical Analysis

All statistical analyses were performed using the R software (version 4.1.0; R Foundation for Statistical Computing, Vienna, Austria). For descriptive statistics, means and standard deviations were calculated for nonparametric data. For categorical variables, numbers and frequencies were described. Mann–Whitney U, Fisher’s exact, and chi-square tests were used to compare the clinical characteristics and outcomes between patients with and without PCCS. Cox proportional analysis was used to identify independent factors associated with 1 year mortality and included all significant predictors in the multivariate analysis, with a *p*-value <0.05. Survival curves were plotted using Kaplan–Meier analysis; survival rates between patients with and without PCCS were compared using the log-rank test, and *p*-values <0.05 were considered statistically significant.

## Results

### Patient Characteristics

A total of 368 patients were included in this study, of whom 105 (29%) had PCCS and 263 (71%) did not. In the PCCS group, 53 patients (50%) underwent surgery for ICM and 52 (50%) for non-ICM. The range of timing between prior cardiac surgery and HT in patients with PCCS varied from 1 week to 44 years, with a median of 45 weeks (IQR 10–132 weeks). Detailed information on the PCCS type is shown in Figure 1.

The demographic data of the patients are shown in Table 1. Most heart failure cases in the PCCS group were due to ICM (50%), whereas dilated cardiomyopathy accounted for 65% of the cases in the non-PCCS group. Patients with PCCS were older, had a lower body weight, and had a higher incidence of cardiopulmonary resuscitation history (17% vs. 9%, *p* = 0.044), pre-transplant ventilator use, and extracorporeal membrane oxygenation (ECMO) support (19% vs. 11%, *p* = 0.039). During surgery, patients with PCCS had significantly longer CPB times than those without PCCS (220 ± 80 vs. 136 ± 49 min, *p* < 0.001). There were no significant differences between the groups regarding sex, blood type, UNOS status, pre-transplant intra-aortic balloon pump use, pre-transplant dialysis, diabetes, renal and liver function, age and body weight of donors, or allograft ischemic time.

**TABLE 1:** Demographic data of recipients with and without PCCS before heart transplantation.

Variate	Overall	Without PCCS	With PCCS	*p*-value
N (%) or mean (±SD)	N = 368	N = 263	N = 105	
Age	45.9 (±16.7)	45.3 (±16.2)	47.5 (±17.9)	0.036
Sex, female	58 (15.8%)	37 (14.1%)	21 (20%)	0.160
Body weight (kg)	62.36 (±17.60)	63.96 (±17.33)	58.34 (±17.70)	0.002
Blood type				0.480
O	101 (27.5%)	67 (25.5%)	34 (32.4%)	
A	121 (32.9%)	86 (32.7%)	35 (33.3%)	
B	109 (29.6%)	83 (31.6%)	26 (24.8%)	
AB	37 (10.1%)	27 (10.3%)	10 (9.5%)	
Etiology				<0.0001
Congenital heart disease	18 (4.9%)	4 (1.5%)	14 (13.3%)	
Ischemic cardiomyopathy	129 (35.1%)	76 (28.9%)	53 (50.5%)	
Dilated cardiomyopathy	184 (50%)	171 (65%)	13 (12.4%)	
Restrictive cardiomyopathy	10 (2.7%)	8 (3%)	2 (1.9%)	
Valvular heart disease	23 (6.3%)	3 (1.1%)	20 (19.1%)	
Others	4 (1.1%)	1 (0.4%)	3 (2.9%)	
UNOS status				0.590
1A	85 (23.1%)	57 (21.7%)	28 (26.7%)	
1B	94 (25.5%)	68 (25.9%)	26 (24.8%)	
2	189 (51.4%)	138 (52.5%)	51 (48.6%)	
Cardiopulmonary resuscitation history	42 (11%)	24 (9%)	18 (17%)	0.044
Pretransplant support				
Ventilator	63 (17%)	39 (15%)	24 (23%)	0.068
IABP	61 (17%)	44 (17%)	17 (16%)	1
ECMO	48 (13%)	28 (11%)	20 (19%)	0.039
Renal dialysis	37 (10%)	23 (9%)	14 (13%)	0.190
Diabetes mellitus	92 (25%)	63 (24%)	29 (28%)	0.510
Hyperlipidemia	80 (22%)	51 (19%)	29 (28%)	0.084
Creatinine	1.4 (±0.8)	1.4 (±0.8)	1.4 (±0.8)	0.880
BUN	30.1 (±17.6)	29.8 (±16.5)	31.0 (±20)	0.980
T-Bil	2.2 (±3.2)	2.3 (±3.5)	2.0 (±2.2)	0.170
In-hospital waiting, days	14.4 (±28.8)	13.1 (±24.0)	17.9 (±38.5)	0.720
Donor				
Age	35.0 (±14.0)	35.5 (±13.9)	33.8 (±14.4)	0.290
Sex, female	106 (29%)	76 (29%)	30 (29%)	1.000
Body weight (kg)	65.0 (±34.6)	63.7 (±14.9)	68.2 (±60.2)	0.640
Allograft ischemia time (min)	161.4 (±63.4)	160.6 (±61.9)	163.39 (±67.4)	0.910
Cardiopulmonary bypass time (min)	155.1 (±66.8)	136.1 (±49.3)	202.3 (±80.2)	<0.0001

PCCS, previous conventional cardiac surgery; IABP, intra-aortic balloon pump; VAD, ventricular assist device.

### Short-Term Outcomes

Patients with PCCS had a higher incidence of post-transplant ECMO support (29% vs. 15%, *p* = 0.003), renal dialysis (41% vs. 21%; *p* < 0.001), and postoperative re-exploration or delayed sternal closure (31% vs. 18%; *p* = 0.004) (Table 2). Although not statistically significant, patients with PCCS also showed a higher incidence of early bloodstream infection and a longer post-transplant hospital stay (18% vs. 11%, *p* = 0.059; 49 ± 55 vs. 38 ± 23 days, *p* = 0.085, respectively).

**TABLE 2:** Short-term outcomes between recipients with and without PCCS.

Post-transplant	Overall	Without PCCS	With PCCS	*p*-value
N (%) or mean (±SD)	N = 368	N = 263	N = 105	
ECMO support	70 (19%)	40 (15%)	30 (29%)	0.003
Renal dialysis^a^	97 (26%)	54 (21%)	43 (41%)	<0.0001
Re-exploration or delayed sternum closure	80 (22%)	47 (18%)	33 (31%)	0.004
Early bloodstream infection (30-day), n (%)	47 (13%)	28 (11%)	19 (18%)	0.059
Hospital stay, days	40.9 ± 35.5	37.7 ± 23.1	48.8 ± 55.0	0.085
In-hospital death	45 (12%)	18 (7%)	27 (26%)	<0.0001
Cause of death, n (% of in-hospital death)				
Primary graft failure	9 (20%)	4 (22%)	5 (19%)	
Infection, sepsis	19 (42%)	8 (44%)	11 (41%)	
Acute rejection	5 (11%)	3 (17%)	2 (7%)	
Aortic rupture	1 (2%)	0	1 (4%)	
Ischemic bowel	3 (6%)	1 (6%)	2 (7%)	
Cerebrovascular event	5 (11%)	0	5 (19%)	
Limb ischemia	2 (4%)	1 (6%)	1 (4%)	
Pulmonary embolism	1 (2%)	1 (6%)	0	

HT, heart transplantation; PCCS, previous conventional cardiac surgery.

^a^ The incidence of renal dialysis included temporal dialysis; 97% of the patients were discharged without dialysis.

The in-hospital mortality rate was significantly higher in patients with PCCS than in those without (26% vs. 7%, *p* < 0.001), and the 1 year mortality rate was also higher in the PCCS group (30% vs. 14%, *p* < 0.001). The leading cause of 1 year mortality in both groups was infection, with cerebrovascular events accounting for a higher proportion in the PCCS group (15.6% vs. 2.6%).

### Risk Factors for One-Year Mortality

Table 3 presents the results of the Cox regression analysis conducted to identify risk factors for 1 year mortality. Univariate analysis revealed several variables associated with 1 year mortality, including recipient age, previous cardiopulmonary resuscitation, UNOS status, pre-transplant ventilator use, pre-transplant intra-aortic balloon pump support, pre-transplant ECMO support, creatinine level, pre-transplant renal replacement therapy, PCCS, donor age, and CPB time. However, in multivariate analysis, only PCCS (hazard ratio (HR) = 2.485, 95% confidence interval (CI) = 1.241–4.975, *p* = 0.01) and CPB time (HR = 1.005, 95% CI = 1.000–1.009, *p* = 0.044) emerged as significant risk factors for 1 year mortality.

**TABLE 3:** Cox regression for risk factors associated with 1-year mortality.

	Univariate			Multivariate		
	Hazard ratio	95% CI	*p*-value	Hazard ratio	95% CI	*p*-value
Age, +1	1.022	(1.005–1.039)	0.011	1.009	(0.987–1.031)	0.441
Sex, male	0.985	(0.518–1.876)	0.964			
BW, + 1 kg	1.000	(0.987–1.013)	0.978			
Blood type (References: AB = 1)						
A	2.151	(0.639–7.240)	0.216			
B	2.869	(0.862–9.557)	0.086			
O	2.504	(0.810–10.876)	0.053			
Smoking	1.272	(0.732–2.210)	0.393			
Hyperlipidemia	1.304	(0.708–2.402)	0.394			
Diabetes	1.627	(0.993–2.665)	0.053			
Previous CPR	3.963	(2.357–6.665)	<0.001	2.539	(0.928–6.947)	0.070
UNOS status (References: 1A = 1)						
1B	0.269	(0.139–0.520)	<0.001	0.380	(0.103–1.402)	0.146
2	0.265	(0.157–0.447)	<0.001	0.597	(0.152–2.347)	0.460
Pre-transplant ventilator	3.751	(2.317–6.074)	<0.001	1.571	(0.396–6.226)	0.520
Pre-transplant IABP	2.869	(1.741–4.727)	0.011	0.925	(0.349–2.452)	0.876
Pre-transplant ECMO	3.582	(2.147–5.976)	<0.001	0.536	(0.144–1.985)	0.350
Pre-transplant dialysis	3.139	(1.773–5.557)	<0.001	2.410	(0.939–6.182)	0.067
Creatinine, + 1 mg/dL	1.295	(1.019–1.646)	0.034	0.963	(0.653–1.418)	0.847
BUN, + 1 mg/dL	1.012	(1.000–1.025)	0.056			
Total bilirubin, + 1 mg/dL	1.019	(0.941–1.103)	0.645			
PCCS (yes)	2.372	(1.482–3.797)	<0.001	2.485	(1.241–4.975)	0.010
Donor age, +1	1.034	(1.016–1.053)	<0.001	1.021	(0.996–1.046)	0.102
Donor sex, male	1.008	(0.600–1.693)	0.975			
Donor BW, + 1 kg	0.999	(0.992–1.007)	0.837			
Allograft ischemic time, + 1min	1.002	(0.998–1.005)	0.376			
CPB time, + 1min	1.006	(1.003–1.009)	<0.001	1.005	(1.000–1.009)	0.044
Etiology of heart failure						
Dilated cardiomyopathy	1					
Congenital heart disease	1.173	(0.356–3.866)	0.794			
Ischemic cardiomyopathy	1.737	(1.037–2.911)	0.036			
Restrictive cardiomyopathy	1.381	(0.328–5.808)	0.66			
Valvular heart disease	2.005	(0.828–4.855)	0.123			
Others	1.826	(0.248–13.443)	0.554			
Etiology and PCCS^a^						
ICM, PCCS (−)	1			1		
ICM, PCCS (+)	4.329	(1.992–9.406)	<0.001	4.848	(1.644–14.299)	0.004
Non-ICM, PCCS (−)	1.347	(0.638–2.845)	0.435	2.447	(0.824–7.267)	0.107
Non-ICM, PCCS (+)	1.739	(0.707–4.280)	0.228	3.554	(1.016–12.439)	0.047

CI, confidence interval; HT, heart transplantation; VAD, ventricular assist device.

^a^ Multivariate analysis was adjusted for age, blood type, diabetes, cardiopulmonary resuscitation, United Network for Organ Sharing status, ventilator use, mechanical circulatory support, donor age, and cardiopulmonary bypass time.

In examining the influence of etiology on heart failure and the various types of surgery, univariate analysis revealed that ICM posed a significant risk factor for 1 year mortality compared to dilated cardiomyopathy (HR = 1.737, 95% CI = 1.037–2.911, *p* = 0.036). Additionally, coronary artery bypass grafting (CABG) was a substantial risk factor for 1 year mortality when compared to patients without prior cardiac surgery (HR = 3.391, 95% CI = 1.934–5.946, *p* < 0.001). As there was a strong correlation between the etiology of heart failure and the type of prior cardiac surgery, a multivariate analysis was conducted, categorizing patients into four groups based on the presence or absence of prior cardiac surgery and the etiology of ICM or non-ICM. After adjusting for other significant factors, the multivariate analysis demonstrated that patients with prior cardiac surgery for ICM had a 4.848-fold increased risk (95% CI = 1.644–14.299, *p* = 0.004), while patients with prior cardiac surgery for non-ICM had a 3.554-fold increased risk (95% CI = 1.016–12.439, *p* = 0.047) compared to those without prior cardiac surgery and non-ICM as the etiology.

### Long-Term Survival

**FIGURE 2:**
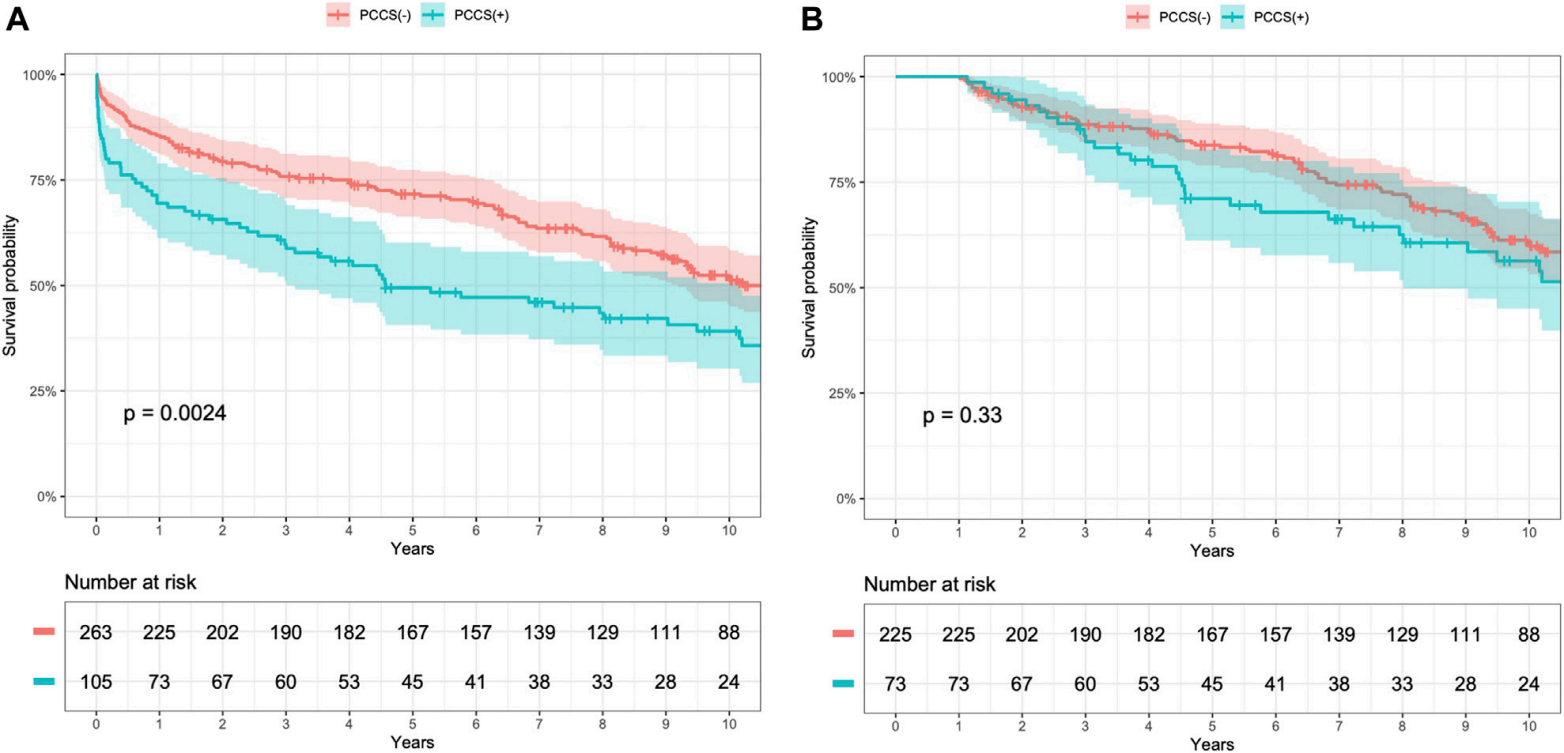
Kaplan–Meier survival curves for patients with and without previous conventional cardiac surgery (PCCS) before heart transplantation (HT) **(A)** and conditional 1 year survival **(B)**.

All patients had complete follow-up data, with a mean follow-up duration of 7.1 ± 5.6 years. Kaplan–Meier survival analysis revealed lower 1, 5, and 10 years survival rates in the PCCS group than in the non-PCCS group (69.5% ± 4.5% vs. 85.6% ± 2.1%, 49.5% ± 5.0% vs. 71.7% ± 2.8%, and 39.2% ± 5.1% vs. 51.8% ± 3.3%, respectively; log-rank test, *p* = 0.0024, Figure 2A). However, excluding patients who died within the first year, the conditional Kaplan–Meier survival curve did not show a significant difference between the PCCS and non-PCCS groups (log-rank test, *p* = 0.33, Figure 2B).

### Subgroup Analysis for Long-Term Survival

**FIGURE 3:**
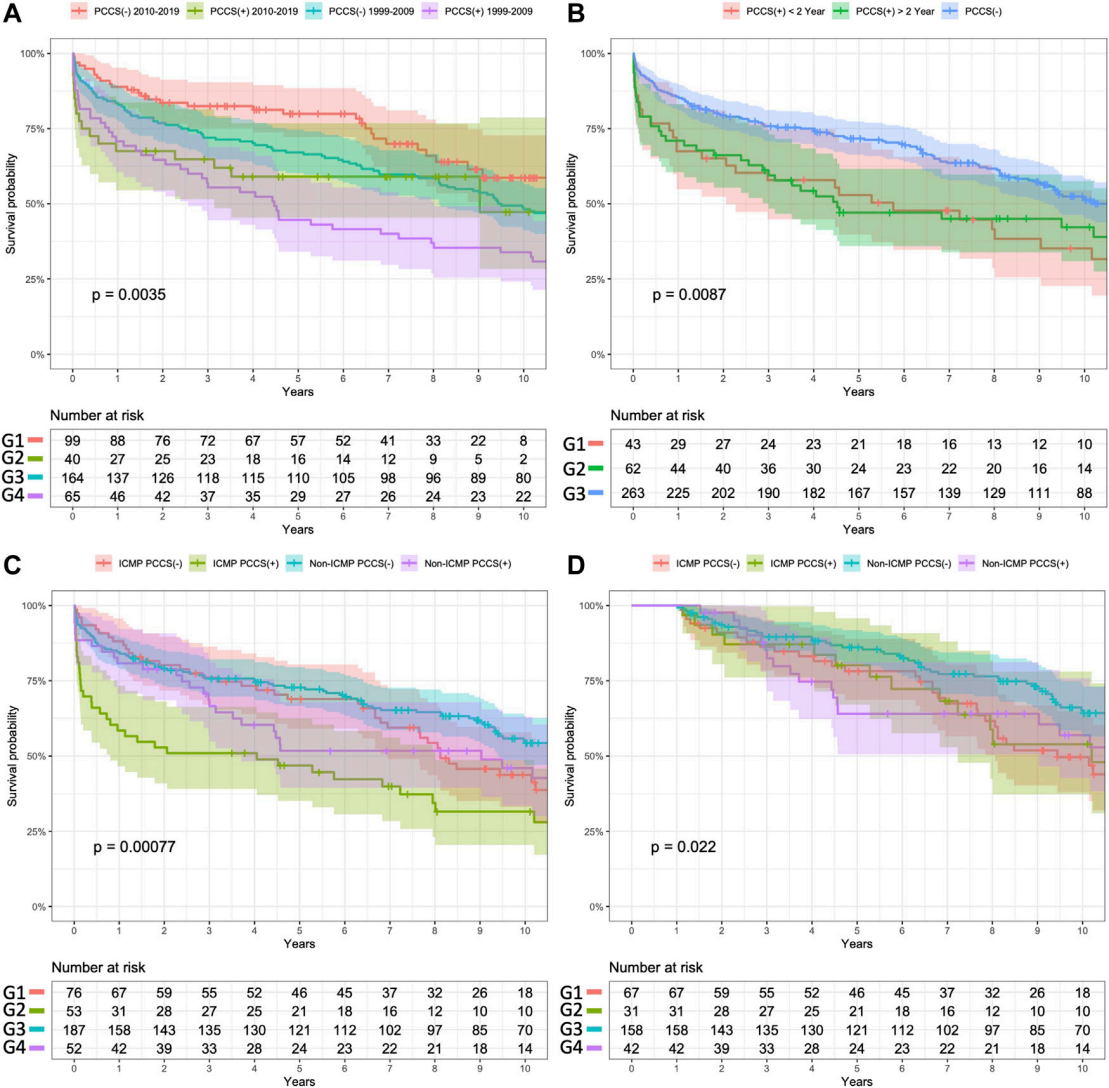
Subgroup analysis of Kaplan–Meier survival curves for study patients. **(A)** Surgery in different eras **(B)**. Prior cardiac surgery within or before 2 years. **(C)** Different purposes, with or without a PCCS. **(D)** Conditional 1 year survival in subgroup analysis. Panel **(A)** Comparison of the survival outcomes between the two groups based on the era of HTs. Patients without PCCS who underwent transplantation after 2010 exhibited better survival outcomes than those in the other three groups (log-rank *p* values for comparisons of G1 vs. G2, G1 vs. G3, and G1 vs. G4 were 0.096, 0.130, and <0.001, respectively). Among patients with PCCS, there was no significant difference in survival outcomes between those who underwent surgery before 2010 (G4) and after 2010 (G2) (log-rank *p*-value = 0.269). Panel **(B)** Segregation of patients with PCCS into two groups based on the timing of PCCS relative to HT. The survival outcome did not differ significantly between these two groups (G1 vs. G2, log-rank *p*-value = 0.103), but their survival was significantly worse than that of patients without PCCS (log-rank *p*-values = 0.026 and *p* = 0.041, respectively). Panel **(C)** Segregation of patients into four subgroups based on the etiology of heart failure and the purpose of PCCS. Patients who underwent PCCS for ICM (G2) had the worst survival outcomes among those in the other subgroups (overall log-rank *p*-value <0.001). Patients who received PCCS for non-ICM (G4) did not show significantly different survival outcomes than those without PCCS (G1 and G3, log-rank *p*-values = 0.485 and 0.346, respectively). As shown in Panel **(D)**, after excluding patients who died within 1 year, the conditional Kaplan–Meier survival analysis showed that even patients without PCCS who had ICM had significantly lower survival outcomes than those without PCCS (G1 vs. G3, log-rank *p*-value = 0.013).

The results of subgroup analyses for long-term survival are shown in Figure 3.

## Discussion

The present study investigated the impact of PCCS on the outcomes of patients undergoing HT, excluding those bridged with a VAD and those who underwent heart re-transplantation. The findings of this study demonstrated that patients with PCCS had significantly poorer short- and long-term outcomes than those without PCCS. In the short term, patients with PCCS had higher rates of post-transplant complications, including the need for renal dialysis, postoperative re-exploration or delayed sternal closure, and post-transplant bloodstream infection. The study revealed that the major survival difference between these two groups occurred in the first year, with PCCS and CPB time during the operation being significant risk factors for 1 year mortality. However, the survival outcome of PCCS did not differ between the new and old eras, and the timing of PCCS and HT did not affect survival. Notably, patients who underwent PCCS due to underlying ICM etiology had significantly poorer 1 year survival, but PCCS did not affect the early survival of patients with non-ICM etiology.

### Early Mortality After HT in Patients With PCCS

The UNOS database report shows that 30% of HTs were performed in recipients with prior cardiac surgery and that prior surgery increased the 1 year and 5 years mortality rates by 1.192 times and 1.104 times, respectively [12, 13, 22]. However, not all types of prior cardiac surgery have the same impact on HT outcomes. Studies indicate that VAD implantation does not affect the subsequent HT outcome, whereas re-transplantation for prior allograft failure exhibits a lower survival rate than other surgery types [11–14]. This study excluded patients with prior VAD implantation or heart re-transplantation to specifically examine the influence of PCCS on HT outcomes. Remarkably, patients with PCCS had a significantly higher 1 year mortality rate (30% vs. 14%) than those without PCCS. PCCS increased mortality risk by 2.485 times within the first year. Notably, the findings of this study highlight the amplified effect of prior cardiac surgery on HT outcomes when exclusively focusing on conventional cardiac surgery, emphasizing the necessity of meticulously considering this factor when selecting HT candidates.

### Cause of Early Death in Patients With PCCS

Infection remained the leading cause of mortality in both groups in this study. Our previous research found that post-transplant dialysis and early bloodstream infection contributes to a 5.5-fold and 3.43-fold increase in early mortality, respectively [18, 21]. In the present study, the PCCS group exhibited a higher incidence of certain factors, namely, post-transplant ECMO support (29%), delayed sternum closure (31%), early bloodstream infection (18%), and dialysis (41%).

While previous studies have shown a correlation between prior cardiac surgery and an increased incidence of acute rejection after HT [6], immune sensitization presents a challenge for patients who have undergone prolonged VAD support or prior HT, which can negatively affect transplant outcomes [15, 16, 23]. However, after excluding these two high-risk groups, our study findings suggest that PCCS may not significantly elevate the risk of acute rejection following HT (7.4% vs. 16.7% in the groups with and without PCCS, respectively).

Interestingly, a significantly higher incidence of cerebrovascular event-related death, including hemorrhagic and ischemic stroke, was observed in the PCCS group (18.5% vs. 0% in the without PCCS group). This finding is consistent with a recent UNOS report that showed a significant increase in post-HT stroke in patients with prior cardiac surgery [24]. Of the five post-transplant strokes observed in this study, two were hemorrhagic, and three were ischemic. Three ischemic strokes occurred in recipients with ICM and prior CABG, who also received ECMO support before HT. ICMP, ECMO support, and re-sternotomy are recognized as the risk factors for post-HT stroke [24–26]. In this study, 50% of patients with PCCS received HT for ICMP, and 19% of patients with PCCS required preoperative ECMO support, which could explain the high incidence of stroke in this group. These findings suggest the importance of further cerebrovascular evaluation before HT, especially in patients with PCCS who require ECMO support and have an underlying ICMP.

### Long-Term Survival in Patients With PCCS

In this study, the 10 years survival rate of patients with PCCS was 39%, significantly lower than that of the patients without PCCS. However, after excluding those who died within a year, the conditional survival analysis showed no significant difference in long-term survival outcomes between the groups. This indicates that the elevated mortality risk associated with PCCS primarily affects the early post-transplant period, consistent with previous studies [3–6, 27].

### Subgroup Analysis for Survival Outcomes

To evaluate the etiology and related surgery on HT outcome, the subgroup analysis revealed that patients with ICM and PCCS had significantly worse outcomes than those in other subgroups during the early postoperative period (Figure 3C). Interestingly, patients with ICM as the etiology but without PCCS showed good short-term survival; however, their long-term outcomes were as poor as those with PCCS. This finding aligns with UNOS reports indicating that ICM, as the etiology of heart failure itself, is a significant risk factor for poor survival after HT [22]. In the context of end-stage heart failure due to ICM, it remains debatable whether patients would benefit more from high-risk conventional bypass surgery for complete revascularization, or from medical treatment with VAD bridging to HT. Further research is needed to resolve this issue. Furthermore, although not statistically significant, patients with PCCS of non-ICM etiology showed worse survival outcomes in the mid-term follow-up (between 3 and 5 years after HT). It is important to consider the impact of different etiologies of heart failure, such as restrictive cardiomyopathy and rheumatic heart disease, on post-transplant outcomes [28–30]. Although our study attempted to address this impact, the limited number of cases prevented us from conducting a comprehensive analysis. Future studies utilizing large databases, such as UNOS reports, could provide more insights into this matter.

Our study also found that both PCCS and extended CPB time were significant risk factors of 1 year mortality. Despite no significant difference in cold ischemic time, patients with PCCS had a total CPB time that was an hour longer. Prolonged CPB can increase micro-emboli formation and the occurrence of renal and neurological complications [31, 32]. The longer duration between PCCS and re-sternotomy may reduce the difficulty of performing re-sternotomy [33]. We found a negative correlation between CPB time and the timing of PCCS and HT (R = −0.19, *p* = 0.05). However, the timing of PCCS to HT did not demonstrate a significant risk reduction in HT outcomes according to the univariate Cox survival analysis. Subgroup analysis also demonstrated that PCCS within or after 2 years did not significantly impact survival outcomes based on Kaplan–Meier analysis (Figure 3B). We found that the era in which HT took place significantly affected survival rates, with patients undergoing HT after 2010 exhibiting better survival outcomes (Figure 3A), thus reflecting advancements in perioperative care and growing HT experience. However, there were no significant differences in survival outcomes between patients with PCCS across different eras. It is important to note that re-sternotomy and prolonged CPB continue to pose challenges for HT in patients with PCCS. Allowing the surgeon sufficient time to perform a demanding re-sternotomy without needing CPB is crucial for improving HT outcomes.

The limitations of this study include the small sample size and the nearly three-decade span it covers, during which significant changes in cardiogenic shock, AKI, and HT management have occurred. While we conducted a thorough examination within our center, it is important to recognize that the findings may have a more localized impact, potentially being more applicable to a single-center scenario rather than offering a comprehensive analysis suitable for a wider range of centers. To better understand the effects of various subgroups, such as the timing of PCCS, type of surgery, and effect of mechanical support (e.g., durable and non-durable VAD support or re-transplantation), a larger number of patients would need to be recruited within a shorter timeframe. This could be achieved through multicenter collaboration or by analyzing a national registry.

Despite these limitations, our study underscores the increased risks and complications faced by patients with PCCS undergoing HT. Therefore, meticulous patient selection and management strategies, including preoperative assessment of re-sternotomy risks, are vital for improving the outcomes. Further research is required to investigate whether the use of VAD in patients with PCCS would improve outcomes after HT and to determine any potential benefits it may offer.

## Data Availability

The raw data supporting the conclusion of this article will be made available by the authors, without undue reservation.

## Abbreviations

CABG, coronary artery bypass grafting; CPB, cardiopulmonary bypass; ECMO, extracorporeal membrane oxygenation; HT, heart transplantation; ICM, ischemic cardiomyopathy; PCCS, previous conventional cardiac surgery; UNOS, United Network for Organ Sharing; VAD, ventricular assist device.

## References

[1] Jasseron C Legeai C Jacquelinet C Nubret-Le Coniat K Flécher E Cantrelle C Optimization of Heart Allocation: The Transplant Risk Score. Am J Transpl (2019) 19:1507–17. 10.1111/ajt.15201 30506840

[2] Laks H Marelli D Fonarow GC Hamilton MA Ardehali A Moriguchi JD Use of Two Recipient Lists for Adults Requiring Heart Transplantation. J Thorac Cardiovasc Surg (2003) 125:49–59. 10.1067/mtc.2003.62 12538985

[3] Uthoff K Wahlers T Cremer J Borst HG. Previous Open Heart Operation: A Contribution to Impaired Outcome After Cardiac Transplantation? Ann Thorac Surg (1997) 63:117–23. 10.1016/s0003-4975(96)00811-9 8993252

[4] Vijayanagar RR Chan GL Weinstein SS. Urgent Heart Transplantation in Patients With Previous Sternotomies. Cardiac Transplant Team. Card Transpl Team. Cardiovasc Surg (1995) 3:331–5. 10.1016/0967-2109(95)93886-t 7655851

[5] Carrel T Neth J Mohacsi P Gallino A Turina MI. Perioperative Risk and Long-Term Results of Heart Transplantation After Previous Cardiac Operations. Ann Thorac Surg (1997) 63:1133–7. 10.1016/s0003-4975(97)00146-x 9124919

[6] Ott GY Norman DJ Hosenpud JD Hershberger RE Ratkovec RM Cobanoglu A. Heart Transplantation in Patients With Previous Cardiac Operations. J Thorac Cardiovasc Surg (1994) 107:203–9. 10.1016/S0022-5223(94)70471-6 8283886

[7] Kawajiri H Manlhiot C Ross H Delgado D Billia F McDonald M High-Risk Cardiac Surgery as an Alternative to Transplant or Mechanical Support in Patients With End-Stage Heart Failure. J Thorac Cardiovasc Surg (2017) 154:517–25. 10.1016/j.jtcvs.2017.03.040 28495061

[8] Finnan MJ Bakir NH Itoh A Kotkar KD Pasque MK Damiano RJ Jr. 30 Years of Heart Transplant:Outcomes After Mechanical Circulatory Support From a Single Center. Ann Thorac Surg (2022) 113:41–8. 10.1016/j.athoracsur.2021.01.064 33675715

[9] Chou NK Chou HW Tsao CI Wang CH Chen KP Chi NH Impact of the Pre-Transplant Circulatory Supportive Strategy on Post-Transplant Outcome: Double Bridge May Work. J Clin Med (2021) 10:4697. 10.3390/jcm10204697 34682819 PMC8539306

[10] Badiwala M Dvirnik N Rao V. Durable Mechanical Circulatory Support as Bridge to Heart Transplantation. Curr Opin Organ Transpl (2022) 27:488–94. 10.1097/MOT.0000000000001012 35950884

[11] Still S Shaikh AF Qin H Felius J Jamil AK Saracino G Reoperative Sternotomy Is Associated With Primary Graft Dysfunction Following Heart Transplantation. Interact Cardiovasc Thorac Surg (2018) 27:343–9. 10.1093/icvts/ivy084 29584854

[12] Axtell AL Fiedler AG Lewis G Melnitchouk S Tolis G D'Alessandro DA Reoperative Sternotomy Is Associated With Increased Early Mortality After Cardiac Transplantation. Eur J Cardiothorac Surg (2019) 55:1136–43. 10.1093/ejcts/ezy443 30649307

[13] Ribeiro RVP Alvarez JS Fukunaga N Yu F Adamson MB Foroutan F Redo Sternotomy Versus Left Ventricular Assist Device Explant as Risk Factors for Early Mortality Following Heart Transplantation. Interact Cardiovasc Thorac Surg (2020) 31:603–10. 10.1093/icvts/ivaa180 33137824

[14] Gaffey AC Phillips EC Howard J Hung G Han J Emery R Prior Sternotomy and Ventricular Assist Device Implantation Do Not Adversely Impact Survival or Allograft Function After Heart Transplantation. Ann Thorac Surg (2015) 100:542–9. 10.1016/j.athoracsur.2015.02.093 26070597

[15] Iribarne A Hong KN Easterwood R Yang J Jeevanandam V Naka Y Should Heart Transplant Recipients With Early Graft Failure Be Considered for Re-Transplantation? Ann Thorac Surg (2011) 92:923–8. 10.1016/j.athoracsur.2011.04.053 21871278 PMC3263700

[16] Miller RJH Clarke BA Howlett JG Khush KK Teuteberg JJ Haddad F. Outcomes in Patients Undergoing Cardiac Retransplantation: A Propensity Matched Cohort Analysis of the UNOS Registry. J Heart Lung Transpl (2019) 38:1067–74. 10.1016/j.healun.2019.07.001 31378576

[17] Engelman DT Ben Ali W Williams JB Perrault LP Reddy VS Arora RC Guidelines for Perioperative Care in Cardiac Surgery: Enhanced Recovery After Surgery Society Recommendations. JAMA Surg (2019) 154:755–66. 10.1001/jamasurg.2019.1153 31054241

[18] Chen JW Chou NK Wang CH Chi NH Huang SC Yu HY Impact of Pretransplant Renal Replacement Therapy on Clinical Outcome After Isolated Heart Transplantation. Transpl Int (2022) 35:10185. 10.3389/ti.2022.10185 35387394 PMC8977403

[19] Fu HY Wang YC Tsao CI Yu SH Chen YS Chou HW Outcome of Urgent Desensitization in Sensitized Heart Transplant Recipients. J Formos Med Assoc (2022) 121:969–77. 10.1016/j.jfma.2021.07.014 34340891

[20] Hsu RB Chang CI Fang CT Chang SC Wang SS Chu SH. Bloodstream Infection in Heart Transplant Recipients:12-Year Experience at a University Hospital in Taiwan. Eur J Cardiothorac Surg (2011) 40:1362–7. 10.1016/j.ejcts.2011.02.033 21459606

[21] Chen JW Chou HW Chou NK Wang CH Chi NH Huang SC Impact of Pre-Transplant Bloodstream Infection on Clinical Outcomes After Heart Transplantation. Transpl Infect Dis (2022) 24:e13834. 10.1111/tid.13834 35427436

[22] Hsich E Singh TP Cherikh WS Harhay MO Hayes D Jr. Perch M The International Thoracic Organ Transplant Registry of the International Society for Heart and Lung transplantation: Thirty-Ninth Adult Heart Transplantation Report-2022; Focus on Transplant for Restrictive Heart Disease. J Heart Lung Transpl (2022) 41:1366–75. 10.1016/j.healun.2022.07.018 PMC10281817 36031520

[23] Kwon MH Zhang JQ Schaenman JM Cadeiras M Gjertson DW Krystal CA Characterization of Ventricular Assist Device-Mediated Sensitization in the Bridge-To-Heart-Transplantation Patient. J Thorac Cardiovasc Surg (2015) 149:1161–6. 10.1016/j.jtcvs.2015.01.003 25702320 PMC7130105

[24] Alvarez P Kitai T Okamoto T Niikawa H McCurry KR Papamichail A Trends, Risk Factors, and Outcomes of Post-Operative Stroke After Heart Transplantation:An Analysis of the UNOS Database. ESC Heart Fail (2021) 8:4211–7. 10.1002/ehf2.13562 34431235 PMC8497374

[25] Patel AR Kuvin JT Pandian NG Smith JJ Udelson JE Mendelsohn ME Heart Failure Etiology Affects Peripheral Vascular Endothelial Function After Cardiac Transplantation. J Am Coll Cardiol (2001) 37:195–200. 10.1016/s0735-1097(00)01057-3 11153738

[26] Cordero Fort A Gavira JJ Alegria-Barrero E Castano S Martin A Ubilla M Prevalence of Metabolic Syndrome in Heart Transplant Patients: Role of Previous Cardiopathy and Years Since the Procedure--The TRACA Study. J Heart Lung Transpl (2006) 25:1192–8. 10.1016/j.healun.2006.06.012 17045931

[27] Aziz T Burgess M Rahman A Campbell C Deiraniya A Yonan N. Early and Long-Term Results of Heart Transplantation After Previous Cardiac Surgery. Eur J Cardiothorac Surg (2000) 17:349–54. 10.1016/s1010-7940(00)00365-1 10773554

[28] DePasquale EC Nasir K Jacoby DL. Outcomes of Adults With Restrictive Cardiomyopathy After Heart Transplantation. J Heart Lung Transpl (2012) 31:1269–75. 10.1016/j.healun.2012.09.018 23079066

[29] Chi NH Chou NK Yu YH Yu HY Wu IH Chen YS Heart Transplantation in End-Stage Rheumatic Heart Disease-Experience of an Endemic Area. Circ J (2014) 78:1900–7. 10.1253/circj.cj-13-1606 24965078

[30] Dan JM Silva Enciso J Lund LH Aslam S. Heart Transplantation Outcomes for Rheumatic Heart Disease: Analysis of International Registry Data. Clin Transpl (2018) 32:e13439. 10.1111/ctr.13439 PMC6384093 30383907

[31] Brown WR Moody DM Challa VR Stump DA Hammon JW. Longer Duration of Cardiopulmonary Bypass Is Associated With Greater Numbers of Cerebral Microemboli. Stroke (2000) 31:707–13. 10.1161/01.str.31.3.707 10700508

[32] Salis S Mazzanti VV Merli G Salvi L Tedesco CC Veglia F Cardiopulmonary Bypass Duration Is an Independent Predictor of Morbidity and Mortality After Cardiac Surgery. J Cardiothorac Vasc Anesth (2008) 22:814–22. 10.1053/j.jvca.2008.08.004 18948034

[33] Fatehi Hassanabad A Zarzycki AN Jeon K Deniset JF Fedak PWM. Post-Operative Adhesions: A Comprehensive Review of Mechanisms. Biomedicines (2021) 9:867. 10.3390/biomedicines9080867 34440071 PMC8389678

